# Medical rehabilitation of older employees with migrant background in Germany: Does the utilization meet the needs?

**DOI:** 10.1371/journal.pone.0263643

**Published:** 2022-02-07

**Authors:** Chloé Charlotte Schröder, Jürgen Breckenkamp, Jean-Baptist du Prel

**Affiliations:** 1 Department of Occupational Health Science, University of Wuppertal, Wuppertal, Germany; 2 Department of Epidemiology & International Public Health, School of Public Health, Bielefeld University, Bielefeld, Germany; University of Hradec Kralove: Univerzita Hradec Kralove, CZECH REPUBLIC

## Abstract

Due to demographic change with an ageing workforce, the proportion of employees with poor health and a need for medical rehabilitation is increasing. The aim was to investigate if older employees with migrant background have a different need for and utilization of medical rehabilitation than employees without migrant background. To investigate this, self-reported data from older German employees born in 1959 or 1965 of the first and second study wave of the lidA cohort study were exploratory analyzed (n = 3897). Subgroups of employees with migrant background were separated as first-generation, which had either German or foreign nationality, and second-generation vs. the rest as non-migrants. All subgroups were examined for their need for and utilization of medical rehabilitation with descriptive and bivariate statistics (chi-square, F- and post-hoc tests). Furthermore, multiple logistic regressions and average marginal effects were calculated for each migrant group separately to assess the effect of need for utilization of rehabilitation. According to our operationalizations, the foreign and German first-generation migrants had the highest need for medical rehabilitation while the German first- and second-generation migrants had the highest utilization in the bivariate analysis. However, the multiple logistic model showed significant positive associations between their needs and utilization of rehabilitation for all subgroups. Further in-depth analysis of the need showed that something like under- and oversupply co-exist in migrant groups, while the foreign first-generation migrants with lower need were the only ones without rehabilitation usage. However, undersupply exists in all groups independent of migrant status. Concluding, all subgroups showed suitable use of rehabilitation according to their needs at first sight. Nevertheless, the utilization does not appear to have met all needs, and therefore, the need-oriented utilization of rehabilitation should be increased among all employees, e.g. by providing more information, removing barriers or identifying official need with uniform standards.

## Introduction

Due to the demographic change and prolonged working lives, the proportion of older employees is increasing in Germany [1, 2] and other European countries, and thus also the number of employees with poor health and functional limitations [3]. Therefore, one major public health goal in the next years and decades should be to avoid premature work exits due to poor health with the help of primary prevention, rehabilitation and occupational re-integration. These will gain relevance in working life, as e.g. medical rehabilitation is aiming at continuous active participation in working life [4]. Additionally, medical rehabilitative services were implemented within several guidelines in Germany over the years, e.g. for coronary heart disease [5]. Consequently, there is a strong expectation that the needs and demands for rehabilitation will increase in the future.

In Germany, in order to be eligible for medical rehabilitation, the objective need must be assessed first. The need for rehabilitation is not automatically officially acknowledged by the psychological or physical impairment, but mainly from the continuing or expected impairment of participation in social and working life [6–8].

The concerned person must submit an application himself, so that individual need can be proven. The validation is jointly done by the rehabilitation providers (e.g. the pension, accident or the health insurance), who coordinate their responsibilities among themselves. Within an objective socio-medical evaluation, information provided by the applicant, doctors, psychological psychotherapists and other therapeutic professions in social work and care are taken into account. However, no uniform procedure to assess the objective need for rehabilitation [9] exists and even the socio-medical evaluations seem to have only limited reliability [10]. Within rehabilitation research several more standardized assessment procedures were suggested to support the identification of the need [7, 11], such as the “Luebecker algorithm” [8], the “Work Ability Index” [12, 13], the “risk index for disability pension” [14, 15] or a “checklist to identify the need for medical rehabilitation by general practitioners" [16, 17]. By now, the latter is also recommended by the northern German pension insurance and provided to general practitioners [17]. Within these assessments, working conditions and exposures and thus the workability are linked to the need for rehabilitation. This is due to certain work exposures increasing the risk of early retirement and disability pension which should be prevented with the help of rehabilitation [18–21]. Therefore, the need for rehabilitation is related to the individual workload.

In particular, groups of employees who have worked as factory workers are burdened by monotonous, repetitive work and physically demanding tasks [18, 19, 21]. In addition, psychosocial workloads (e.g. low scope in decision-making, job insecurity, conflicts at work, time pressure) are suspected to have an influence on the short and long-term probability of early retirement due to illness [20].

Compared to those without a migrant background (non-EMB), employees with a migrant background (EMB), especially foreign nationals, are more frequently exposed to such health-endangering working conditions which our own data has also shown [22–25]. Compared to non-EMB, EMB more often work as manual workers (semi-skilled and unskilled workers), i.e. they often work in low-skilled occupations and have less completed vocational training [22, 24]. Additionally, EMB are more frequently exposed to psychological workloads like lower influence at work which all in all results in lower workability, significantly longer periods of sick leave, more frequent occupational accidents and diseases (e.g. noise-induced hearing loss) [22, 23, 25]. Additionally, when these unfavorable working conditions accumulate over working life, employees in higher working age might even be at higher risk for negative health outcomes [18, 26]. However, the mentioned results mainly apply to foreigners or first-generation migrants, as we found in former analyses, second-generation migrants seem close to natives [22, 25, 27]. Former research in this field likewise showed that descendants of immigrants have fewer differences to the native population, probably due to adaption and different coping processes while growing up in the host country. Reasons might be e.g. that they were not exposed to the whole migration process themselves and have a higher utilization of social network as a coping method compared to first-generation groups [22, 28–30].

The group of EMB comprises employees born outside of Germany (first-generation, G1) and employees born in Germany, but with one or both parents born abroad (second-generation, G2) [2]. They can have German or foreign nationality, although the second-generation mainly has German nationality. Their proportion in the working population is continuously increasing and has risen from 16.2% in 2010 to 24.4% in 2019. From these working EMB, 37.4% were > 45 years old in 2019, so part of the older working population [1, 2].

Based on these circumstances for foreigner, which are mostly G1 EMBs, one would assume that they are likely to have a higher need for rehabilitation. So far, there are no studies investigating this issue in EMBs or generally in the working population in Germany, as there is no gold standard to assess the need for rehabilitation in Germany, yet.

Those with foreign nationality, are more likely to retire earlier due to disability, compared to employees with German nationality [23]. Such differences may be attributed to occupational and health factors, but also to lower utilization of health services such as medical rehabilitation. Until 2018, studies showed that people with a migrant background are less likely to utilize medical rehabilitation compared to those without (non-EMB) [27, 31–33], possibly due to barriers such as lack of information, language problems, illiteracy, cultural aspects etc. [33–35]. However, there were no differences found in studies published in 2018 or later, so findings are inconsistent and often lack information about the second-generation, because of the limited differentiation of migrant background [33].

This lacking differentiation is a major limitation of other previous studies on migrants’ work, health or utilization of rehabilitation services in Germany. This is because quantitative studies are often based on the analysis of secondary data such as process data. In such data sets, it is mostly the feature “nationality” that allows for the differentiation of the migrant background. Yet, in Germany, such a definition leads to the misclassification of about half of all people with a migrant background as non-migrants, as 11.1 million of a total 21.2 million people with a migrant background, had German nationality in 2019 [2]. Additionally, primary studies often do not make any further differentiation between migrant groups, even when other operationalizations than nationality are used [33]. However, EMB are a heterogeneous group and should be investigated in more detail.

To our knowledge, even representative studies in Germany investigating the need for rehabilitation in older employees are missing and likewise for subgroups with migrant background. Furthermore, it is highly important to investigate the utilization of rehabilitation depending on the need, to assess if the provision of health services like medical rehabilitation meets the needs and demands in general.

Therefore, the current study aimed to primarily investigate if subgroups of EMB have a different need for rehabilitation than non-EMB and secondly, if they use rehabilitation divergently when considering their respective need for rehabilitation.

## Materials and methods

### Study design and participants

The prospective lidA (leben in der Arbeit) cohort study investigates work, health and employment in older employees of two age cohorts (1959, 1965) as part of the “babyboomer generation” in Germany. This study is based on a representative two-stage random sample of all socially insured employees of these cohorts in Germany in 2009. Due to the sampling specification, sworn civil servants and self-employed were not included. The participants were interviewed at home for each assessment wave by computer assisted personal interviews (CAPI), including a variety of questions about health, private life and work, as the participants get closer to retirement. The baseline survey took place in 2011 (N = 6585), the second wave in 2014 (N = 4244) and the third wave in 2018 (N = 3586).

All procedures performed in this study involving human participants were in accordance with the ethical standards of the institutional and/or national research committee and with the 1964 Helsinki declaration and its later amendments or comparable ethical standards. Informed verbal consent was obtained from all individual participants included in the study after informing about study content, procedures and data protection in writing, according to good epidemiological practice. This procedure has been approved by the Ethics Committee of the University of Wuppertal (dated from 05/12/2008 and 20/11/2017, MS/BB 171025 Hasselhorn). The ethics approval refers to the whole lidA cohort study, not only this partial study. All the lidA data was anonymized before starting analyses. A more detailed description of the lidA cohort study including power calculation etc. can be found elsewhere [36].

Results of attrition analysis showed an almost selection-free realization of the sample in relation to the sociodemographic characteristics used in the cited analyses [37–39] for all waves. However, a more differentiated analysis revealed attrition of 65% for low educational level in foreign and 63% in German G1 EMB compared to about 42% in non-EMB and 43% in G2 EMB. Since this analysis included data from the first and second study wave, we performed inverse probability weighting for subgroups of migrant status and educational level. The sample was restricted to those employed at least 1h/week in both study waves (N = 3961, unweighted). Due to the weighting, cases with missing values in migrant background or educational level were also excluded. Consequently, the final sample consisted of 3897 individuals.

### Operationalization

#### Dependent variable

The outcome of the study was the self-reported “utilization of medical rehabilitation” indicated in the second study wave. Participants were asked to report whether they had utilized an in- or outpatient rehabilitation service in the previous three years. The answers for in- and outpatient services were summarized vs. no utilization of rehabilitation, generating a binary variable.

#### Independent variable

The counterpart and other aspect of rehabilitation was the “need for medical rehabilitation*”*, which we operationalized with the help of a summarizing score taking different relevant aspects of life into account. A range of such variables was considered in a checklist in a study by Deck et al. and is now recommended by the northern German pension insurance for general practitioners to assess the need for rehabilitation [16, 17]. This checklist provided the basis for the summarizing score, so that representative and appropriate variables of the lidA study were assigned to each category of the checklist (see Table 1). All those self-reported variables were taken from the first study wave to consider need for and utilization of rehabilitation consequentially over the course of time. If any of the mentioned variables applied to a person, then the item got the coding 1. At the end, there was a possible range of values from 0 to 15, while summing up at least 10 valid items and allowing 5 missing items. The score correlated significantly with general health, the single item Short Form-12 Health Survey (SF-12) [40], by r_pbis_ = .568.

**Table 1:** Variables used in the lidA study, categorized according to the checklist of Deck et al. [16].

Original category of the checklist	Assigned variables of the lidA-study (self-reported)
Indication of rehabilitation: disease requiring treatment, chronification of disease, comorbidities	• Incidence of disease requiring treatment (in the last 12 months)
	• declared handicap/disability
Functional limitations: impairments in daily or working life	*•* poor physical health (lowest tertile of the SF-12 physical health scale, version of the socio economic panel survey) [40, 41]
	• frequent limitation due to pain (in the last 4 weeks) in daily life or at work
Accompanying psychological symptoms: depressiveness, anxiety, exhaustion	*•* poor mental health (lowest tertile of the SF-12 mental health scale, version of the socio economic panel survey) [40, 41]
Influenceable risk factors: nicotine abuse, alcohol, lack of exercise, obesity, dyslipidemia	• BMI > 30, BMI = weight/(height*2)
	• less/no sports or exercise in leisure time
	• regular smoking at time of survey
Therapy: outpatient therapy not sufficient or not available nearby, intensification required, unfavorable working hours	• working hours that are unfavorable for therapy (such as shift work, especially night and alternating shifts)
Adverse influences in work, profession and everyday life: significant physical or environmental work exposure e.g. heavy lifting, noise etc., psychological stress	• lower workability in relation to physical and mental job demands (second dimension of the workability index, >8 points: normal work ability, <8 points: low work ability) [42]
	• high work stress (highest tertile of the effort-reward-imbalance ratio, indicating high efforts but low rewards) [43, 44]
	• more than one physical work exposure (e.g. heavy lifting and carrying; for at least half of the working time)
Disability: current or threatened incapacity for work, long or repeated sick leave in the last 2 years	• official sick leave > 30 days (in the last 12 months)
	• officially declared reduced capacity to work or job-related incapacity
	• indication of "prolonged illness" in the question about employment
Motivation and disease management: motivation to participate and to change own lifestyle is present, own disease management strategies are insufficient	no variables from the first or second wave of the lidA-study can be assigned

#### Migrant background

The lidA cohort study allows to distinguish between migrant groups by means of different specific indicators as proposed by Schenk et al. [45]. EMB were defined based on the participants’ self-reported country of birth and nationality and on the country of birth of each of their parents. Participants born in Germany, with German nationality and with both parents being born in Germany constitute the reference group (non-EMB). The group of EMB was divided in three subgroups to investigate potential differences. Firstly, they were separated in generations, based on a definition provided by the German Federal Statistical Office [1, 2], so into first-generation (G1 EMB) and second-generation (G2 EMB), as described before. Secondly, G1 EMB were divided into those with German and foreign nationality, as in own (unpublished) pre-analyses, differences between these groups were detected. In G2 EMB nearly all participants had German nationality, so these weren’t differentiated any further. In the end, there were four groups: non-EMB vs. German G1 EMB, foreign G1 EMB and G2 EMB.

#### Covariates

To control for sociodemographic differences, the following variables were considered as potential confounders when comparing groups with different migration background regarding the association of their need for and utilization of rehabilitation: Year of birth (1959/1965), sex (male/female), and education. Education was operationalized with a score combining school and professional education according to the recommendations of the German Society of Epidemiology for the measurement and quantification of sociodemographic characteristics in epidemiological studies [46]. Accordingly, values from 1 (= not any graduation) to 8 (= school leaving examination and graduation from college) were calculated for each combination of school and professional education. For ease of interpretation the score was classified in three categories: high, medium and low level education.

### Statistical analysis

Due to group differences in attrition between the first and the second study wave relating to migrant status and educational level, basic inverse probability weighting was used to account for potential non-response bias. Inverse probability weighting is a method, where the data is standardized on a certain population, which is different from the one, in which the data was collected [47]. In our case the data was standardized on the population of the lidA baseline assessment in 2011. For each subgroup the equation was: weight = percentage in wave 1/percentage in wave 2, so e.g. for the group of non-EMB with low education: 19.71%/17.89% = 1.1017. Simultaneously, the weighting factors were calculated for all other subgroups, which can be found in S1 Table. All reported results are based on weighted analyses; however, in the S2 Table unweighted characteristics are additionally presented for comparison.

Descriptive and bivariate statistics including chi-square tests, F-tests within analyses of variance (ANOVA) and a Tukey post-hoc test were used to characterize the full sample and specifically investigate differences between groups. For the multiple logistic regression analyses, possible multicollinearities were determined as a pre-check using linear regression models of the independent variable, utilization of rehabilitation. The results of the linear regression analyses are not shown because no statistical evidence of multicollinearity was found. The inflation of variance for all variables was ≤ 1.09. Tests for possible interactions of the need with the sociodemographic covariates were done, which were all not statistically significant. Finally, multiple logistic regressions were performed to investigate the influence of the need for the utilization of medical rehabilitation for each migrant group separately. To further control for sociodemographic differences, the logistic regressions were adjusted for sex, year of birth and education in the full model.

In all statistical tests p-values (two-tailed) <.05 were considered to be statistically significant. These statistical analyses were performed using SPSS version 25.0 (IBM Corp.).

In addition, average marginal effects (AMEs) were computed for all logistic regressions with SAS 9.4. They allow to compare the results of nested models that otherwise may be biased by unobserved heterogeneity. The AME shows for each variable in a regression model how much the event probability changes when the independent variable increases by one unit, or rather when a binary independent variable changes its level [48]. All multiple analyses were done as complete case analyses.

For interpretational purposes additional exploratory analysis were done to examine the utilization depending on the need in more detail for each subgroup separately on bivariate level.

For this, the need score was divided into tertiles, in order to see the percentage of utilization in people with lower, medium or higher need.

## Results

### Descriptive and bivariate analysis

In Table 2 the characteristics of all participants included in the analyses are presented, shown as weighted results (n = 3897). Most of the participants were non-EMB (82.4%), around 7% each German G1 EMB and G2 EMB, and the smallest group was foreign G1 EMB with 3.3%. Due to deliberate oversampling, participants born in 1965 were overrepresented in all subgroups. The same applied to female sex in all groups, the proportion of women was always higher than for men. The distribution of educational level differed significantly between the groups (p<.001), nearly half of foreign G1 EMB had low educational level (45.4%) while the other groups had percentages between 23.7% (non-EMB) and 30.4% (German G1 EMB). However, they also had the highest proportion of high educational level with 25.4% compared to the rest with around 20%.

**Table 2:** Characterization of study population (weighted sample^a^, n = 3897).

	Non-EMB (n = 3211)	German G1 EMB (n = 276)	Foreign G1 EMB (n = 130)	G2 EMB (n = 280)	p-value^b^
Sex [n (%)]					
Male	1481 (46.1)	129 (46.7)	61 (46.9)	121 (43.2)	.800
Female	1729 (53.9)	147 (53.3)	69 (53.1)	159 (56.8)	
Year of birth [n (%)]					
1959	1458 (45.4)	135 (48.9)	50 (38.5)	117 (41.8)	.152
1965	1753 (54.6)	141 (51.1)	80 (61.5)	163 (58.2)	
Education level [n (%)]					
High	663 (20.6)	55 (19.9)	33 (25.4)	61 (21.8)	< .001
Medium	1787 (55.7)	137 (49.6)	38 (29.2)	141 (50.4)	
Low	761 (23.7)	84 (30.4)	59 (45.4)	78 (27.9)	
Utilization of rehabilitation [n (%)], m = 3					
Yes	390 (12.2)	48 (17.4)	14 (10.8)	48 (17.1)	.009
No	2818 (87.8)	228 (82.6)	116 (89.2)	233 (82.9)	

EMB, employees with migrant background; G1, first-generation; G2, second-generation; m, number of missing values due to respondents not responding to the item, from weighted results.

^a^ Total case numbers of each variable vary slightly because of rounding after weighting.

^b^ tested with Chi^2^-test.

Concerning the outcome of utilized rehabilitation, significant differences were likewise observed (p = .009). The highest utilization was reported by German G1 EMB and G2 EMB (around 17% respectively) and the lowest by foreign G1 EMB (10.8%) and non-EMB (12.2%).

**Fig 1:**
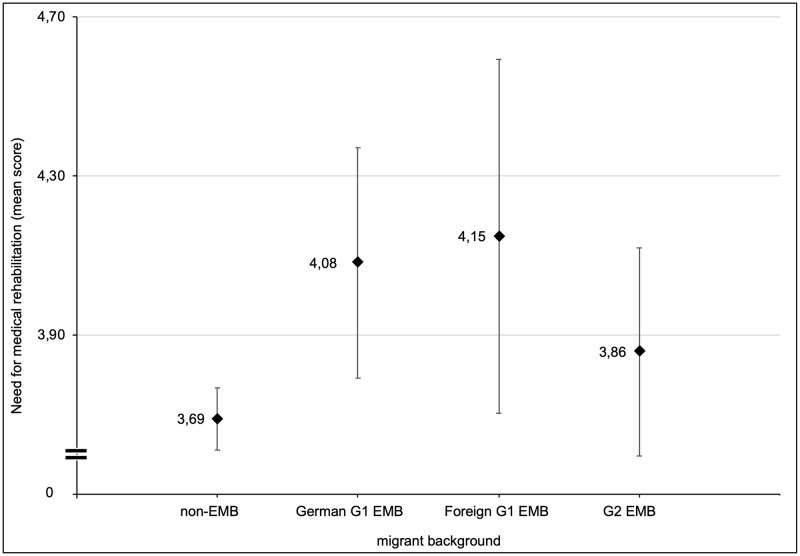
Arithmetic mean values and 95%-confidence intervals of the need score for rehabilitation in migrant groups (weighted results, n = 3897).

In contrast, foreign G1 EMB showed the highest need for rehabilitation when comparing means of the need score (Fig 1). The mean values differed significantly between the four groups as determined by one-way ANOVA [F(3, 3978) = 5.91, p <. 001, η^2^ = 0.004]. A post-hoc test revealed that the need for rehabilitation was statistically higher for German G1 EMB (4.14 ± 2.48, p = 0.006) and foreign G1 EMB (4.23 ± 2.63, p = 0.043) compared to non-EMB (3.66 ± 2.25). There was no statistically significant difference between G2 EMB and non-EMB (p = 1.0).

### Multiple logistic regressions

To answer the second research question, logistic regressions were conducted separately for each migrant group to investigate further behavioral or migrant-group-specific differences (see Table 3). In bivariate analyses, foreign G1 EMB showed the highest need for rehabilitation (see Fig 1), but the lowest utilization of rehabilitation (see Table 2). To examine the association for each group, the odds and the probability for using rehabilitation were calculated depending on the need score. In all models, the need was positively associated with the utilization of rehabilitation in each group: the higher the need, the more likely the utilization.

**Table 3:** Stratified logistic regressions for the utilization of rehabilitation services depending on the need for rehabilitation and further sociodemographic variables (weighted results).

	Crude model: need	Full model: need + sex, year of birth, education
**Non-EMB (n = 3208/ n**_**events**_ **= 390)**		
OR (95% CI)	1.24 (1.19–1.30)***	1.25 (1.19–1.31)***
AME	+0.0228	+0.0234
R^2^	0.052	0.054
**German G1 EMB (n = 276/ n**_**events**_ **= 48)**		
OR (95% CI)	1.22 (1.08–1.38)***	1.25 (1.10–1.43)***
AME	+0.0273	+0.0289
R^2^	0.058	0.084
**Foreign G1 EMB (n = 127/ n**_**events**_ **= 14)**		
OR (95% CI)	1.65 (1.27–2.13)***	2.02 (1.40–2.91)***
AME	+0.0318	+0.0421
R^2^	0.276	0.353
**G2 EMB (n = 279/ n**_**events**_ **= 48)**		
OR (95% CI)	1.27 (1.11–1.46)***	1.30 (1.12–1.50)***
AME	+0.0310	+0.0327
R^2^	0.070	0.087

* p < .05,

**p < .01,

*** p < .001.

AME, average marginal effects; CI, confidence interval; M, Model; n_events_, number of events where the outcome = 1 in the logistic regression; OR, Odds Ratio; p, p-value; Ref., Reference; R^2^, Nagelkerke pseudo-R^2^.

In the model adjusted for sex, year of birth and education, foreign G1 EMB had the highest odds (OR 2.02, 95% CI 1.40–2.91) and the highest probability (4.2% for each unit change) to utilize medical rehabilitation when the need increased. The other groups showed ORs from 1.25 to 1.30 each. When testing narrowed models, in detail only one sociodemographic control variable at a time to follow the “one in ten rule”, nearly no change in the coefficients was detected.

### Additional exploratory analysis for interpretation

Our bivariate analyses revealed that foreign G1 EMB have a higher need but lower utilization (Tables 1 and 2), which was presumed before analysis. To be able to interpret this apparent contradiction, we examined the utilization depending on the need in more detail for each subgroup separately on bivariate level (Table 4). Hereby, undersupply for all subgroups independent from migrant background was detected to the extent that over 70% of the people with higher need are not utilizing rehabilitation services. However, in three groups there are still 7–10% of those with lower need that have used rehabilitation in the past. Only in foreign G1 EMB those with lower need have not used rehabilitation at all.

**Table 4:** Utilization of rehabilitation services depending on the need for rehabilitation (separated for each group, weighted results, row percent, n = 3894).

	Need (tertiles)	Utilization		p-value^a^
		No	Yes	
**Non-EMB(n = 3208)**	Lower	92.4%	7.6%	< .001
	Medium	90.4%	9.6%	
	Higher	80.5%	19.5%	
**German G1 EMB (n = 276)**	Lower	89.2%	10.8%	.001
	Medium	89.0%	11.0%	
	Higher	71.6%	28.4%	
**Foreign G1 EMB (n = 130)**	Lower	100%	0%	.011
	Medium	90.5%	9.5%	
	Higher	80.0%	20.0%	
**G2 EMB (n = 280)**	Lower	92.8%	7.2%	.009
	Medium	82.7%	17.3%	
	Higher	75.8%	24.2%	

EMB, employees with migrant background; G1, first-generation; G2, second-generation.

^a^ tested with Chi^2^-test.

## Discussion

In the present study, we analyzed the need for and respective utilization of rehabilitation for employee groups with and without migrant background. For the primary research question, we identified that foreign and German G1 EMB had the highest need for rehabilitation when measuring with our need score. The highest utilization of rehabilitation was reported by German G1 EMB and G2 EMB with 17%, while foreign G1 EMB showed the lowest with 11%. Secondarily, when considering the respective need in multiple logistic regressions, significant positive associations with the utilization were found for each subgroup. Foreign G1 EMB showed the highest association between need and utilization.

However, the results of foreign G1 EMB have to be carefully interpreted as the case number of this group was quite low (Table 3), as well as the number of “utilized rehabilitation” within multiple logistic regression. This is due to loss-to-follow up between the first and second study wave, which was weighted for, but also due to lower participation rate among foreigners in general in the first study wave. If the case numbers had been higher, also for German G1 EMB and G2 EMB, differences between confidence intervals (CI) of the four groups would probably have been more precise, as the CI would get narrower. However, based on the confidence interval of the OR in the adjusted model, our study only showed significant differences between foreign G1 EMB and non-EMB while foreign G1 EMB have higher probability to utilize medical rehabilitation than non-EMB when the need increased.

This analysis is the first in Germany to identify the need for rehabilitation in different migrant groups compared to non-migrants. The findings of a higher need for rehabilitation in G1 EMB and especially in foreign G1 EMB match our assumptions before analysis as this group often experience unfavorable working conditions, as mentioned in the introduction. Our results showed, when assessing need with the help of our need score: the higher the need, the higher the utilization of rehabilitation for all groups. However, further barriers for instant utilization of healthcare and rehabilitation e.g. language problems, illiteracy or cultural aspects might exist for foreign G1 EMB due to own migration experiences and potential different health beliefs within their cultural background. Hence, when assessing need for medical rehabilitation with our need score, this group showed zero utilization of rehabilitation when having low need, compared to the other groups in Table 4. They might only utilize rehabilitation when really necessary and their health situation is worsening.

In more detail, our additional analysis for interpretational purposes detected that over 70% of the people with higher need did not utilize rehabilitation services but instead 7–10% of those with lower need used rehabilitation in the past. Only those with lower need did not use rehabilitation at all in foreign G1 EMB. This raises the question as to whether health services like medical rehabilitation are truly authorized according to the need in Germany. Especially in older working age, health services like medical rehabilitation should be provided depending on the existing need. Only in this way, equal opportunities to stay healthy and actively in work and prevent early exit can be assured and unnecessary costs avoided. While there have been some projects in Germany in the past, aiming at improving the information about and access to medical rehabilitation where needed [e.g. 49], according to our result further efforts would be worthwhile. The accessibility to medical rehabilitation in general might be improved by further information campaigns or reducing formal access barriers (e.g. application process, waiting times, travel distances or charges for those with lower/no income) with diversity in mind. More migrant-specific strategies to reduce language or cultural barriers would be important as well.

However, the main dependent factor is, of course, the instrument to assess the need for rehabilitation, as the term “need” is not distinct and results are highly dependent of the chosen instrument. As described before, there are several operationalizations within rehabilitation research in Germany suggested to support the identification of need. In the presented study, we decided to orientate the operationalization towards the checklist of Deck et al. [16], as it covers various life aspects of the person affected: Incidence of disease, functional limitations, psychological factors, other risk factors such as smoking, motivation and coping with the disease, therapy, inability to work and impairments in work and everyday life. It convinced us that the checklist is nowadays recommended by the northern German pension insurance for general practitioners to assess the need for rehabilitation [17]. In our case however, the items of the need score were based on subjective information of the study participants and not on an objective assessment of the need for rehabilitation. Nonetheless, the items were collected independently and without the purpose of assessing the need for rehabilitation. Such a checklist or scoring not only helps general practitioners to screen their patients, but could additionally help the official need assessment within the socio-medical evaluation,

In the future, the operationalization should definitely be standardized and so we are calling for a harmonization of the assessment procedure for the need of rehabilitation as other rehabilitation researchers [7, 8]. As rehabilitation is oriented towards the biopsychosocial model of illness and health, which is the basis of the International Classification of Functioning, Disability and Health (ICF) [50, 51], this could be another approach for a standardized need assessment. It is already stated in the social code IX in Germany that the determination of the need for rehabilitation should be carried out by an instrument that is based on the ICF, while also considering different life aspects e.g. mobility, domestic life, communication etc. [52]. Besides, research has already found out that the ICF Generic 6 score is a valid tool to assess functioning in several clinical settings [53], so this could be another possible instrument to use for need assessment. To our knowledge, further research is still going on to implement the ICF in other settings and test its practicability and reliability there [e.g. 54–57].

Another important aspect, especially for the further outlook, constitutes the timing of needs assessment, as people with need should be identified and allocated early enough to medical rehabilitation. Schlöffel and colleagues [58] tested an intervention of a web-based self-test to identify need for rehabilitation and subsequently the effectiveness on the application rate. The self-test was based on WAI and IMET (“Index to measure restrictions of participation”). Though, this intervention showed no significant effect as the only means, as Spanier and Bethge also investigated [49, 59]. A solution could be to combine different means. Bethge and his team already proposed in 2012 [12] a 3-staged procedure with screening of register data using a validated risk index at first [15], then postal screening with WAI for persons with high risk in the first step and lastly giving them consultation and information for the application for rehabilitation. This procedure would be more likely to improve application rates [58] and the utilization of rehabilitation according to personal need in the long run. However, this procedure is not implemented yet within the German pension insurance or other rehabilitation providers, as far as we are informed.

### Strengths & limitations

Our study has several strengths. First, the sample is representative for the German population of socially insured employees of the considered two age cohorts [37, 38]. Second, the lidA study has the strength to differentiate more detailed subgroups with migrant background. Different indicators to map migrant status are used as recommended by Schenk et al. [45], not only nationality. Another strength is the variety of the study characteristics, so that several important factors to measure the need of rehabilitation could be taken into account. Here, validated instruments like WAI or SF12 were used to represent the different areas of life which are considered in the checklist of Deck et al. [16]. In earlier rehabilitation research these were already associated with the need for rehabilitation, however it remains an open question whether the summing score to assess the objective need for rehabilitation is the right instrument, as there is no gold standard in Germany so far. Another advantage was the ability to consider the need for and utilization of rehabilitation in logical time order, as the need was assessed from the first study wave and the utilization in the second study wave. Of course, no causality can be proven in a study like this.

Despite these strengths, the study also has its limitations. First, there is no gold standard for the assessment of the need for medical rehabilitation in Germany. So, we could not test the validity of our assessment instrument derived from the check list suggested by Deck et al. (2009) for general practitioners more comprehensively. Furthermore, certain aspects which influence the official need were discussed in previous rehabilitation research, but could not be considered, as there were no suitable items within the first and second lidA study wave assessed. These include the overall rehabilitation prognosis, the participant’s motivation and therapy options, such as the local infrastructure for rehabilitation. Yet, these factors might be more relevant in estimating the long-term success of medical rehabilitation and to a lesser degree in assessing the actual need for rehabilitation, which was the focus of our investigation. Overall, the items of the need score were based on subjective information of the study participants and not on an objective assessment of the need for rehabilitation. Nonetheless, the items were collected independently and without this purpose. Another limitation is the restriction to two age cohorts within socially insured employees due to sampling, where the lidA study does not include sworn civil servants and self-employed persons. While the majority of employees in Germany are socially insured [2, 36], employees with migrant background are underrepresented in the group of civil servants and overrepresented in the group of self-employed. So, the health status of migrants and non-migrants could be different if civil servants and self-employed were included. Consequently, the findings of this study are limited to socially insured employees born in 1959 or 1965. Yet, overall there are at least comparable percentages of different migrant backgrounds in the lidA-study in comparison to the German microcensus [2]. Further restriction to generalizability could have been introduced by language bias through the conduction of the study in German, where EMB were potentially excluded when having language problems. Finally, as mentioned before, the case number of foreign G1 EMB was quite low so that the results for this group have to be interpreted carefully.

## Conclusions

According to our results and operationalizations, all subgroups showed suitable use of rehabilitation according to their needs at first sight, foreign G1 showed the highest association. However, when looking more in detail, something like under- and oversupply co-exist in all subgroups, while foreign G1 employees with lower need were the only ones without rehabilitation usage. Yet, undersupply exists in all groups independent of migrant status. Therefore, the need-oriented utilization of rehabilitation should be increased among all employees, e.g. by providing more information, removing barriers or identifying official need with the same standards. The findings highlight the necessity for distinct operationalization and investigation of migrant groups in research and resulting policy.

## Supporting information

S1 Table Weighting factors for inverse probability weighting. (DOCX)

S2 Table Characterization of study population (unweighted sample, n = 3944). (DOCX)
