# Correction: Medical rehabilitation of older employees with migrant background in Germany: Does the utilization meet the needs?

**DOI:** 10.1371/journal.pone.0272325

**Published:** 2022-07-26

**Authors:** Chloé Charlotte Schröder, Jürgen Breckenkamp, Jean-Baptist du Prel

The following information is missing from the Funding statement: We acknowledge support from the Open Access Publication Fund of the University of Wuppertal.
