# Supplementary material for: Medical rehabilitation of older employees with migrant background in Germany: Does the utilization meet the needs?
Source: PLoS One. 2022 Feb 7;17(2):e0263643. doi: 10.1371/journal.pone.0263643 (PMC8820604; doi:10.1371/journal.pone.0263643)
Supplement: S1 Table — (DOCX) [file pone.0263643.s001.docx]

**S1 Table. Weighting factors for inverse probability weighting**

|  | Non-EMB | German  G1 EMB | Foreign  G1 EMB | G2 EMB |
| --- | --- | --- | --- | --- |
| Education level |  |  |  |  |
| High | 0.9297 | 1.0440 | 1.0890 | 0.8631 |
| Medium | 0.9439 | 1.1984 | 1.3696 | 0.8929 |
| Low | 1.1017 | 1.7169 | 1.8487 | 1.1300 |
