# Supplementary material for: Medical rehabilitation of older employees with migrant background in Germany: Does the utilization meet the needs?
Source: PLoS One. 2022 Feb 7;17(2):e0263643. doi: 10.1371/journal.pone.0263643 (PMC8820604; doi:10.1371/journal.pone.0263643)
Supplement: S2 Table — (DOCX) [file pone.0263643.s002.docx]

**S2 Table. Characterization of study population (unweighted sample, n=3944).**

|  | Non-EMB  (n=3317) | German  G1 EMB  (n=229) | Foreign  G1 EMB  (n=98) | G2 EMB  (n=300) | p-value^a^ |
| --- | --- | --- | --- | --- | --- |
| Sex [n (%)] |  |  |  |  |  |
| Male | 1520 (45.8) | 105 (45.9) | 45 (45.9) | 129 (43.0) | .827 |
| Female | 1797 (54.2) | 124 (54.1) | 53 (54.1) | 171 (57.0) |  |
| Year of birth [n (%)] |  |  |  |  |  |
| 1959 | 1501 (45.3) | 113 (49.3) | 37 (37.8) | 125 (41.7) | .154 |
| 1965 | 1816 (54.7) | 116 (50.7) | 61 (62.2) | 175 (58.3) |  |
| Education level [n (%)] |  |  |  |  |  |
| High | 718 (21.7) | 54 (24.5) | 31 (33.7) | 72 (24.0) | < .001 |
| Medium | 1896 (57.3) | 116 (52.7) | 29 (31.5) | 159 (53.0) |  |
| Low | 693 (21.0) | 50 (22.7) | 32 (34.8) | 69 (23.0) |  |
| Utilization of rehabilitation [n (%)], m=3 |  |  |  |  |  |
| Yes | 402 (12.1) | 38 (16.6) | 11 (11.2) | 52 (17.3) | .018 |
| No | 2912 (87.9) | 191 (83.4) | 87 (88.8) | 248 (82.7) |  |
| EMB, employees with migrant background; G1, first-generation; G2, second-generation; m, number of missing values due to respondents not responding to the item, from weighted results.  ^a^ tested with Chi^2^-test. | | | | | |
